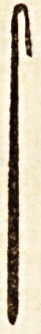# On the Formation of Artificial Pupil, without Injury to the Crystalline Lens or Its Capsule

**Published:** 1835-01-01

**Authors:** Frederick Tyrrell

**Affiliations:** Surgeon to St. Thomas's Hospital, and to the London Ophthalmic Infirmary, Moorfields.


### *On the Formation of Artificial Pupil, without injury to the Crystalline Lens or its Capsule, by* FREDERICK TYRRELL, Esq. Surgeon to St. Thomas's Hospital, and to the London Ophthalmic Infirmary, Moorfields.

AMONG the numerous cases of blindness from obstruction, obliteration, or destruction of the central aperture of the iris, (called the pupil,) there are very many in which the crystalline lens, or its investing membrane, are affected, when any operation to effect restoration of vision must necessarily implicate these structures; but there are also many instances in which the lens and its capsule retain their original integrity and perfection.

Hitherto, as far as my inquiries and observation have enabled me to ascertain, there has not been any plan devised by which the surgeon could form an artificial pupil, without at the same time injuring the crystalline body; so that, if it were previously sound, it would be rendered opake by an operation for forming an artificial pupil, and consequently its removal would be necessary, to make the artificial pupil serviceable.

The loss of this highly important structure is not the only evil resulting from injury to it, for such injury often produces consequences fatal to the formation of the new pupil itself, and is sometimes destructive to vision altogether.

Thus the opake lens, or some fragments of it, by pressing on the iris, often create and maintain an inflammatory action in the iris, under which the artificial opening closes; or the inflammation thus excited extends to the deeper seated tissues, (as the choroid and retina,) and produces such changes as destroy their functions, and occasion organic amaurosis.

It is my intention, in the present paper, to describe an operation which I have practised for several years, and of the value of which I now feel confident, as it has enabled me to avoid the evils I have described. It leaves the lens and its capsule uninjured; it creates little risk of inflammation; and the restoration of vision by it is in most cases perfect, without any artificial aid, which must be resorted to when the lens has been destroyed.

First, I shall describe the cases to which the operation is applicable; and, secondly, explain the mode of performing the operation.

An aperture penetrating the cornea, whether created by wound or by ulceration, immediately permits the escape of the aqueous fluid, and is usually followed by a prolapse of the iris; and if the aperture be large, so much of the iris is sometimes protruded that the pupil becomes exceedingly diminished or destroyed; and in the reparation of the mischief, the iris becomes adherent to the cornea, and embedded in the cicatrix, constituting *synechia anterior*. In the cases in which the pupil is nearly destroyed the remaining part is often useless, in consequence of the portion of the cornea which covers it becoming opake in the process, by which the injury to the cornea is repaired.

When the opacity of the cornea is irremediable, and the remaining part of the pupil is so small that the influence of belladonna cannot increase it, an operation is as necessary to restore vision, as in those cases in which the pupil has been entirely destroyed from a similar cause.

When under these circumstances, one third or more of the cornea retains its transparency and natural character, and the subjacent portion of the iris is of healthy aspect, the patient will probably have a distinct perception of light, and a perfect result may then be anticipated from operation; but when less than one fourth of the cornea is free from disease, a less favourable termination to operation may be expected: we may hope to restore useful, but not perfect vision.

When the iris is discoloured or dull, it has been inflamed, and it may be adherent to the anterior capsule of the lens, and the capsule itself be opake. The evidence of previous inflammation of the iris, therefore, renders the prognosis of the case, as regards the result of an operation, very doubtful; but it should not deter the surgeon from its performance, for I have restored good or useful vision when I have had little prospect of success.

If the patient can distinguish light from darkness, I always consider the operation warrantable, as affording a chance of benefit, even when the features of the case are otherwise most unfavourable.

The operation is then applicable to all cases in which the pupil has been entirely destroyed, or so nearly so as to be useless, in consequence of prolapse of the iris, provided that a portion of the cornea remains clear, and the patient has a perception of light.

It sometimes happens that the natural pupil becomes obscured, and rendered almost useless, by the formation of a dense and large central opacity of the cornea, as the result of cicatrization of an ulcer, or from the effect of an escharotic, and the opacity is permanent. In such a case vision may be improved or made good, by altering the position of the original pupil, which can be readily effected by the operation I am about to describe.

The instruments required in the performance of the operation are, first, a knife or needle, to make an aperture in the cornea of sufficient size to admit of the introduction of a hook. As it is desirable that the opening in the cornea should not be much larger than is sufficient to admit the hook, I prefer a needle of a diameter rather larger than the hook, to a knife.

Secondly, a hook of the form delineated in the margin; the extremity of the curved part of this instrument must be perfectly smooth.

In forming an artificial pupil on the nasal side, I have found it necessary to have the stem of the instrument bent nearly at a right angle, to enable me to avoid the nose.

Thirdly, a fine pair of scissors, to cut off the portion of the iris to be brought out of the opening in the cornea by the hook.

Fourthly, a pair of fine dissecting forceps.

The operator has not usually much choice of position for making the artificial pupil, as it must of course be formed under the transparent part of the cornea, which is frequently very limited; but he should at all times, as far as circumstances will admit, form it towards the inferior part of the cornea, vision downwards being of much greater service than upwards.

During the operation I prefer having the patient recumbent, with the head slightly raised, and with the light falling rather obliquely on the face. Then seating myself at the head of the patient, so that his head rests against the lower part of my sternum, I can command the superior eyelid of either eye, and hardly require the aid of an assistant.

In elevating and fixing the superior palpebra, the forefinger should be placed near the centre of its free margin, so that the extremity of the finger touches the surface of the globe, and the cilia are pressed outwards: by very moderate force the lid can then be elevated, but, in effecting this, the forefinger must not only press against the eyebrow or superciliary ridge, but also slightly against the surface of the globe, or the eyelid will probably evert. At the same time the point of the middle finger should be placed on the globe, near the inner canthus.

The left hand should be used to the right eye, and the right hand to the left eye, when the surgeon occupies the position described; but, if not ambidexter, he must change his position, and employ an assistant when operating upon the left eye.

By well regulated pressure with the points of the two fingers, the surgeon can in great measure command the motions of the globe, in most instances, and thereby facilitate the operation.

The superior lid being elevated and fixed, and the globe under command, the needle is to be passed through the cornea, close to its junction with the sclerotic, at the point previously determined upon; it should freely enter the anterior chamber.

On withdrawing the instrument, a part or the whole of the aqueous fluid usually escapes; and, if the iris has been previously free and unaffected, (as in the simple case of dense central opacity of the cornea,) a small part of it may prolapse; and I always endeavour, by firm pressure with the fingers placed on the globe, to effect such a protrusion.

A prolapse of the iris, however trifling, immediately occasions an alteration in the figure and in the position of the pupil; and thus, the puncture of the cornea being followed by a spontaneous protrusion of the iris, sometimes effects all that is desired in such a case, by bringing the pupil under a transparent part of the cornea.

If under these circumstances the prolapse of the iris be not sufficient, the projecting part should be seized by a fine pair of forceps, and drawn from the wound, until sufficient has been pulled out to effect the desired change in the position of the pupil.

Supposing that the iris does not prolapse when the anterior chamber has been opened, in the case of dense central opacity, or when the operation is resorted to, in consequence of the original pupil being nearly destroyed, the further steps of the operator will be similar.

The patient being allowed a few moments' rest after the use of the needle, the upper eyelid should be again elevated as before, and the hook be carefully passed through the opening effected by the needle into the anterior chamber, and carried on, until the point enters the pupil. In passing the hook, the curved part should be kept towards the cornea, until the extremity has entered the pupil, when the instrument should be rotated, so that the hook may receive the free margin of the iris.

The iris being caught by the hook, the instrument should be steadily withdrawn, and when the curved part arrives at the aperture in the cornea, if it is arrested, the instrument must be again rotated, to turn the curved part towards the cornea, otherwise there may be some difficulty in getting it out, in consequence of the extreme part of the hook catching against the inferior edge of the aperture. The hook brings out a portion of the iris, and produces an immediate alteration in the position of the natural pupil, or an enlargement of that previously diminished by disease; and by gradually withdrawing the instrument from the aperture, a further portion of the iris may be generally pulled out, until the desired effect on the pupil is produced. The protruding portion of the membrane may then be removed close to the aperture in the cornea by the scissors. If the hook tears through the part of the iris which is brought out of the anterior chamber by it before a sufficient portion has been drawn out, the protruded part should be seized with the forceps, and a further portion withdrawn and cut off.

Supposing that the original pupil has been entirely destroyed, it is necessary to make a small aperture in the iris as well as in the cornea, for the passage of the hook; and this is the only respect in which the operation in such a case would differ from that described. The opening in the iris should be made by the same instrument by which the cornea is penetrated; and, if a needle be employed, the opening in the iris can be effected without increasing the size of that in the cornea. After therefore the needle has entered the anterior chamber, the point should be very carefully directed to the part at which the iris and cornea are adherent, and passed through the former close at its junction with the latter; a very small opening suffices. In puncturing the iris the operator must direct the point of the needle to the cornea, for there is usually in these cases so little space of posterior chamber, that the capsule of the lens may be easily injured if the point of the needle be passed at all backwards. The subsequent steps of the operation in such a case should be as I have already described.

If in the performance of the operation an effusion of blood takes place into the anterior chamber from the torn iris, it of course obscures all view of the opening effected; the surgeon should then be content to separate as much of the iris as he considers requisite, provided it can be done easily; but, in case of any difficulty, I would strongly recommend him to leave the case, and complete the work by a second operation, after the eye has perfectly recovered from the effects of the first.

Under mild antiphlogistic treatment, and cooling applications, the effused blood becomes rapidly absorbed.

This evil most frequently happens when the iris has been previously inflamed, and a recurrence of iritis is to be dreaded as the effect of the operation, or from the pressure of the extravasated blood. I therefore usually give mercury, unless there be some cause generally to forbid its use, for the purpose of checking the iritis, and promoting the absorption of the effused blood. The early exhibition of mercury in moderate doses, once or twice in the day, will always expedite the absorption of the blood, and in my opinion often tends to prevent the development of iritis.

When the artificial pupil exposes an opake lens or capsule, it should be left until the patient has quite recovered from the operation for artificial pupil, and be afterwards removed by the use of the needle.

After the artificial pupil has been formed by considerable laceration of the iris, the belladonna has little or no influence.

I have very frequently employed it, but have never seen any decided effect from it; the orbicular fibres of the iris being in great measure destroyed, the radiating fibres contract, and remain afterwards fixed, the opposing power being lost.

I think it unnecessary to illustrate the paper by the introduction of cases, as I do not believe that they would render my explanation more distinct. The operation has been performed by me so frequently at the London Ophthalmic Infirmary, and at St. Thomas's Hospital, and with such beneficial results, that there is sufficient testimony of its merits.

My object, in offering this paper for publication, is to contribute my mite to the advancement of one of the most interesting branches of surgery which has within a few years been rescued from obscurity and empiricism, and, under scientific cultivation, is rapidly attaining a degree of perfection scarcely surpassed in any other department.

17, *New Bridge Street, Blackfriars;*

*December,* 1834.

## Figures and Tables

**Figure f1:**